# A Comparative Study on the Dietary Iodine Intake and the Contribution Rates of Various Foods to the Dietary Iodine Intake among Residents in Zhejiang in 2010 and 2022

**DOI:** 10.3390/nu16183101

**Published:** 2024-09-14

**Authors:** Jiaxin He, Lichun Huang, Simeng Gu, Zhe Mo, Danting Su, Chenyang Liu, Fanjia Guo, Yuanyang Wang, Zhijian Chen, Ronghua Zhang, Xiaoming Lou, Guangming Mao, Xiaofeng Wang

**Affiliations:** 1 Department of Environmental Health, Zhejiang Provincial Center for Disease Control and Prevention, 3399 Binsheng Road, Hangzhou 310051, China; jxhedyx@163.com (J.H.); smgu@cdc.zj.cn (S.G.); zhmo@cdc.zj.cn (Z.M.); fjguo@cdc.zj.cn (F.G.); yywang@cdc.zj.cn (Y.W.); zhjchen@cdc.zj.cn (Z.C.); xmlou@cdc.zj.cn (X.L.); 2 Department of Nutrition and Food Safety, Zhejiang Provincial Center for Disease Control and Prevention, 3399 Binsheng Road, Hangzhou 310051, China; wlch12688@163.com (L.H.); sdting12368@sina.com (D.S.); zhrh128@163.com (R.Z.); 3 School of Public Health, Zhejiang University, Hangzhou 310058, China; 22118893@zju.edu.cn

**Keywords:** dietary iodine, coastal, contribution rate, iodized salt

## Abstract

We aim to analyze the changes in dietary iodine intake and the contribution rates of various foods to it after the reduction in salt iodine concentration in Zhejiang. We used data from two cross-sectional nutrition surveillance surveys conducted by the Zhejiang Provincial Center for Disease Control and Prevention in 2010 (9798 residents) and 2022 (5980 residents). In both surveys, multi-stage stratified and systematic sampling were adopted, and uniformly trained investigators conducted the dietary surveys using a 24 h dietary review and weighing record methods for 3 consecutive days. From 2010 to 2022, the median salt iodine concentration and the consumption rate of qualified iodized salt in Zhejiang households dropped from 28.80 to 22.08 mg/kg and from 76.65% to 64.20%, respectively. Moreover, the residents’ median dietary iodine intake decreased from 277.48 to 142.05 μg/d. Significant interregional differences in dietary iodine intake were found in 2010 and 2022 (*H* = 639.175, *p* < 0.001; *H* = 588.592, *p* < 0.001, respectively); however, no significant differences existed between urban and rural areas (*p* > 0.05). From 2010 to 2022, the proportion of residents with dietary iodine intake below the estimated average requirement increased from 15.10% to 34.80%, while that of residents with intake above the tolerable upper limit decreased from 15.00% to 2.90%. The contribution rate of salt to dietary iodine intake among residents in Zhejiang decreased from 74.92% to 48.54%, showing an apparent overall downward trend despite the dietary intake being generally adequate (markedly inadequate in coastal regions). The salt iodine concentration and the consumption rate of qualified iodized salt in households in Zhejiang showed downward trends. Salt remained the main source of dietary iodine; however, its contribution decreased significantly. Zhejiang may need to reverse the trend of the continuous decline in the consumption rate of qualified iodized salt to protect the health of its residents.

## 1. Introduction

Iodine is an essential trace element for the human body. It plays a vital role in the synthesis of thyroid hormones, which are involved in regulating metabolism and are indispensable for the healthy growth of the body [1] and normal growth and development of the brain and central nervous system [2,3]. Iodine intake is closely related to human health. Insufficient iodine intake can lead to goiter and other adverse health consequences [4,5]. In contrast, excessive iodine intake is associated with increasing thyroid dysfunction [6,7]. Therefore, a balanced diet is essential to maintain normal thyroid function. Iodine deficiency and excess can lead to a range of diseases [4,5,6,7]; therefore, assessing the iodine status of a population is a requisite public health initiative.

Iodine concentrations in food grown in different regions differ because of the varied distribution of iodine on the earth’s surface. Approximately 80% of iodine ingested by humans comes from food, 10–20% from drinking water, and <5% from the air [8]. Marine products, such as marine algae, kelp, shellfish, and marine fish, are important sources of iodine in nature [9]. In many parts of the world, the iodine intake from food is too low to meet the physiological needs or the estimated average requirement (EAR). Therefore, the World Health Organization recommends fortifying salt with iodine as the preferred strategy for preventing iodine deficiency [10]. Consuming iodized salt is a safe and effective means of iodine supplementation. It has the advantages of simple implementation, low cost, and effortlessness for long-term compliance, which is the best way to supplement iodine [11].

As a key indicator for assessing the iodine nutrition status of the global population, dietary iodine intake has long attracted extensive attention from the international scientific research community. Using dietary surveys, many scholars have explored the iodine intake in different regions and populations to reveal the current iodine nutrition status, identify potential risks of iodine deficiency and excess, and provide an empirical basis for formulating scientific and rational iodine intervention strategies. Sun et al. [12] revealed that in the United States, females aged < 14 years, between 15 and 49 years, and >50 years (including non-pregnant and non-lactating women) showed a downward trend in iodine intake between 2011 and 2020. The dietary frequency survey method used by Hou et al. [13] revealed that iodine intake through salt was a major component (61.97%) of the total iodine intake of adults in Tianjin, China. In contrast, food products from marine algae accounted for only 11.04% of the total iodine intake despite their high iodine concentrations, and aquatic foods had a lower contribution rate to the total iodine intake. Gordon et al. [14] conducted a randomized, placebo-controlled, double-blind trial in 184 children aged 10–13 years in Dunedin, New Zealand. They found that supplementing iodine can improve perceptual reasoning abilities in children with mild iodine deficiency. However, due to geographical differences, dietary iodine intake varies between regions.

Since the universal salt iodization (USI) strategy was implemented in 1995, the iodine nutritional status in China has greatly improved [15]. According to the National Food Safety Standard–Iodine Content in Edible Salt (GB 26878-2011) [16], China reduced the iodine concentration in iodized salt in 2012. In the same year, Zhejiang lowered the iodine concentration in iodized salt from 35 mg/kg (±30%) to 18–33 mg/kg, which had been the case for 10 years until 2022. With the introduction of the Reform Plan for the Salt Industry System [17], the variety of salt available has increased, and non-iodized salt has gradually entered the market. In recent years, the consumption rate of qualified iodized salt among residents in Zhejiang has decreased significantly, and the iodine nutrition status in the province has also changed. Therefore, clarifying the dietary iodine intake and the source of dietary iodine among the population in Zhejiang may facilitate the formulation of a scientific iodine supplementation strategy.

In this study, we aimed to analyze the relationship between salt and iodine intake in the Zhejiang population and estimate the actual contribution rates of different food sources to iodine intake. We analyzed the dietary iodine intake, the contribution rate of different food sources to iodine intake, and their trends and possible influencing factors among residents based on the data of two cross-sectional nutrition surveillance surveys in Zhejiang in 2010 and 2022. Our findings may promote a more comprehensive and accurate understanding of the current iodine supplementation among the Zhejiang population and serve as a basis to implement better the strategy of “adapting measures to local conditions, targeted guidance, and scientific iodine supplementation” to eliminate iodine deficiency disorders (IDDs), prevent iodine excess, and protect the health of residents.

## 2. Materials and Methods

### 2.1. Study Population

We used the dietary survey data of two cross-sectional nutritional surveillance surveys conducted by the Zhejiang Provincial Center for Disease Control and Prevention in 2010 and 2022. According to the administrative divisions, geographical locations, and economic levels of the regions in Zhejiang, and the relevant situation of IDDs, 22 survey counties (cities or districts, including 11 urban and rural survey sites each) and 16 survey counties (cities or districts, including eight urban and rural survey sites each) in the province were selected by multi-stage stratified sampling in 2010 and 2022, respectively. In 2010, three townships (subdistricts) were selected in each survey county (city or district) according to geographical location, one administrative village (residents’ committee) was chosen as the survey site in each selected subdistrict or township, and 50 households were selected by systematic sampling at each survey site, comprising 9798 study participants. In 2022, three townships (subdistricts) were selected in each survey county (city or district) according to geographical location, two administrative villages (residents’ committees) were chosen as survey sites in each selected township (subdistrict), and 30 households were selected by systematic sampling at each survey site, comprising 5890 study participants. In 2010, the Ethics Committee of Zhejiang Academy of Medical Sciences approved the study, and all participants provided informed consent. In 2022, the Ethics Committee of the Zhejiang Provincial Center for Disease Control and Prevention approved the study (Approval No. 2022-018-01; Date: 10 May 2022), and all participants provided informed consent.

### 2.2. Protocol for the Estimation of Sodium and Iodine Intake

#### 2.2.1. Survey Content (Same for 2010 and 2022)

Dietary status survey: The survey plan and questionnaire were designed based on the 2002 Survey on Nutrition and Health Status of Chinese Residents. The survey comprised basic information and dietary surveys. The basic information survey included household size, general information (age, ethnicity, marital status, education, occupation), and dietary patterns and habits. The dietary survey adopted the 24 h dietary review method to survey the individual food intake of all permanent members of the survey household for 3 consecutive days; specifically, each respondent answered questions about all foods (including staple and non-staple foods, snacks, fruits, and drinks) consumed by the respondent within 24 h for 3 consecutive days (2 working days + 1 rest day), and the answers were recorded. The consumption of various edible oils, monosodium glutamate, salt, and other condiments was investigated for 3 consecutive days using the weighing record method.

Drinking water iodine survey: Samples of drinking water were collected in each selected resident’s committee (village). If the survey site was located in a centralized water supply area, one sample each of finished and drinking water was collected from each of the two selected households. If the survey site was in a decentralized water supply area, one drinking water sample each from the source and the residents’ homes were collected in the eastern, western, southern, northern, and central areas of the survey site. Each sample was at least 25 mL. The water supply method and well depth were recorded.

Household salt iodine survey: Edible salt samples were collected from all survey households, mixed properly, and sealed in plastic bags. The samples were taken from the salt jars used daily by the residents of the home. Each sample was at least 30 g.

#### 2.2.2. Laboratory Testing

Water iodine: In 2010 and 2022, water iodine concentrations were tested according to the “Water Iodine Detection Methods Suitable for Iodine-Deficient and -Excess Areas” (recommended by the National Reference Laboratory for Iodine Deficiency Disorders) [18].

Salt iodine: In 2010, the iodine concentrations in unfortified salt samples were measured by direct titration, whereas the iodine concentrations in Sichuan salt and other fortified salt samples were measured using the arbitration method (GB/T 13025.7-1999) [19]. In 2022, the iodine concentrations in the salt samples were measured according to the General Test Method for Salt Industry–Determination of Iodine (GB/T 13025.7-2012). In contrast, the iodine concentrations in Sichuan and seaweed salt samples were detected by direct titration. In addition, salt samples with an iodine concentration of <5 mg/kg were remeasured using the redox method [20].

The criteria for judging the main analytical indicator were assessed as follows:

Salt iodine: The salt samples were evaluated according to the National Food Safety Standard–Iodine Content in Edible Salt (GB 26878-2011) and the amount of iodine added to edible salt specified by the former Zhejiang Provincial Department of Health. Salt samples with iodine concentration < 5 mg/kg were considered non-iodized; salt samples with iodine concentration < 18 mg/kg or >33 mg/kg were considered unqualified iodized; and salt samples with an iodine concentration between 18 and 33 mg/kg were considered qualified iodized [16].

Dietary iodine intake: The recommended iodine intakes are based on the Dietary Reference Intakes for Chinese Residents [21] (Table 1).

Distribution of survey regions: In 2010, the coastal regions included Ningbo, Zhoushan, Taizhou, and Wenzhou Cities; the sub-coastal regions included Hangzhou, Jiaxing, Huzhou, and Shaoxing Cities; and the inland regions included Jinhua, Quzhou, and Lishui Cities. In 2022, the coastal regions included Dinghai District, Jiaojiang District, Sanmen County, Cixi City, and Lucheng District; the sub-coastal regions included Wuxing, Linping, Fuyang, and Fenghua Districts, and Haining City; and the inland regions included Suichang County, Changshan County, Shengzhou City, Jindong District, Dongyang City, and Yongjia County.

#### 2.2.3. Calculation and Analysis of Dietary Iodine Intake and Contribution Rates of Different Food Sources to the Total Dietary Iodine Intake

Calculation of dietary iodine intake: Dietary iodine intake mainly comes from food, drinking water, and salt, and the calculation formula is dietary iodine intake = ∑ (*C_i_* × F*C_i_*), where *C_i_* is the iodine concentration in food, drinking water, and salt and F*C_i_* is the consumption of food, drinking water, and salt. The food types in the study were classified as kelp, *Porphyra*, marine fish, and other foods.

Calculation of contribution rate: The contribution rate of a certain type of food to the total dietary iodine intake was based on the percentage of the average daily iodine intake from that type of food to the average daily total dietary iodine intake.

#### 2.2.4. Data Source

The Dietary Survey of Nutrition Surveillance data in Zhejiang conducted in 2010 and 2022 were used for food and salt consumption data, respectively. The iodine intake of drinking water was based on the Dietary Reference Intakes for Chinese Residents. The iodine concentration of food was based on the second and sixth Chinese Food Composition tables [22,23], respectively. The iodine concentration of drinking water and salt was based on the Zhejiang provincial iodine nutrition survey results in 2010 and 2022, respectively.

### 2.3. Statistical Analysis

Data were analyzed using SPSS version 21.0 (IBM Corporation, Armonk, NY, USA). Data normality was tested using the Kolmogorov–Smirnov test, and the non-normally distributed continuous data are expressed as *M* (*P_25_*, *P_75_*). Multiple independent samples, such as regions, were compared using the Kruskal–Wallis *H* test, and two independent samples, such as urban/rural areas and sex, were compared using the Mann–Whitney *U* test. The Nemenyi test was used for pairwise comparisons. Count data are expressed as rates and proportions, and the chi-squared test was used for intergroup comparisons. The chi-squared trend test was used for trend analysis. For a two-sided test, the significance level was α = 0.05.

## 3. Results

### 3.1. Basic Information

The 2010 survey included 9798 residents, including 4752 (48.50%) males and 5046 (51.50%) females (Table 2). Of these, 4949 (50.50%) were urban residents and 4849 (49.50%) were rural residents.

The 2022 survey included 5890 residents, including 2734 (46.40%) males and 3156 (53.60%) females. Of these, 3128 (53.10%) were urban residents and 2762 (46.90%) were rural residents (Table 2).

### 3.2. Consumption of Iodized Salt in Zhejiang

The consumption rates of qualified iodized salt in Zhejiang in 2010 and 2022 were 76.65% and 64.20%, respectively (Table 3). The median (*P_25_*, *P_75_*) iodine concentration of salt consumed by households in Zhejiang decreased from 28.80 (22.93, 32.40) mg/kg in 2010 to 22.08 (0.00, 24.51) mg/kg in 2022 (*Z* = −43.28, *p* < 0.001). Under the category of urban/rural areas and distribution of survey regions, the iodine concentrations of salt consumed by households in Zhejiang differed significantly between 2010 and 2022 (all *p* < 0.001).

### 3.3. Dietary Iodine Intake of the Residents in Zhejiang

The median (M) (interquartile range: *P_25_*, *P_75_*) dietary iodine intake of the residents in Zhejiang decreased from 277.48 (155.50, 430.66) μg/d in 2010 to 142.05 (58.94, 237.11) μg/d in 2022 (*Z* = −42.408, *p* < 0.001) (Table 4). Under the categories of urban/rural areas, distribution of regions, and whether iodized salt was consumed, the iodine concentrations of salt consumed by households in Zhejiang differed significantly between 2010 and 2022 (all *p* < 0.001).

In 2010 and 2022, there were significant differences in dietary iodine intake among different regions (*H* = 639.175, *p* < 0.001 and *H* = 588.592, *p* < 0.001, respectively) and between people who consumed iodized salt and those who did not (*Z* = −38.501, *p* < 0.001 and *Z* = −52.246, *p* < 0.001, respectively); however, urban and rural areas in Zhejiang did not differ significantly in both surveys.

### 3.4. Relationship between Dietary Iodine Intake and Reference Intake

In 2010, the dietary iodine intake did not reach the EAR in 15.10% (1478/9798) of the residents and exceeded the tolerable upper limit (UL) in 15.00% (1466/9798) (Table 5). In contrast, in 2022, the dietary iodine intake did not reach the EAR in 34.80% (2050/5890) of the residents and exceeded the tolerable UL in only 2.90% (171/5890). These results indicate that the composition ratio of the relationship between dietary iodine intake and reference intake among residents in 2010 differed significantly from that in 2022 (*χ*^2^ = 1501.409, *p* < 0.001).

In 2010, the composition ratio of the relationship between dietary iodine intake and reference intake among residents differed significantly among different regions (*χ*^2^ = 1209.798, *p* < 0.001). The proportion of residents with dietary iodine intakes below the EAR in different regions was ranked as follows: coastal regions (30.80%) > sub-coastal regions (6.70%) > inland regions (5.40%) (*χ*^2^_trend_ = 849.457, *P_f_*_or trend_ < 0.001). In 2022, the composition ratio of the relationship between dietary iodine intake and reference intake among residents was significantly different among different regions (*χ*^2^ = 723.632, *p* < 0.001). The proportion of residents with dietary iodine intakes below the EAR in different regions was ranked as follows: coastal regions (54.80%) > sub-coastal regions (30.20%) > inland regions (16.00%) (*χ*^2^_trend_ = 647.777, *P*_for trend_ < 0.001).

### 3.5. Sources of Dietary Iodine for the Residents in Zhejiang

In 2010, salt, drinking water, and various foods contributed 73.45%, 1.39%, and 25.16%, respectively, of the dietary iodine intake (Table 6). In 2022, salt, drinking water, and various foods contributed 48.54%, 8.84%, and 42.62%, respectively, of the dietary iodine intake. Compared with 2010, the contribution rates of salt and *Porphyra* to dietary iodine intake in 2022 decreased significantly (all *p* < 0.001). In contrast, the contribution rates of drinking water, kelp, other seafood, and other foods (excluding *Porphyra*) increased significantly (all *p* < 0.01).

Under the categories of distribution of regions and urban/rural areas, the contribution rates of salt and drinking water to dietary iodine intake differed significantly between 2010 and 2022 (all *p* < 0.001).

## 4. Discussion

In this study, the trends and contribution rates of different food sources to dietary iodine intake among residents in Zhejiang were studied using dietary surveys. The survey results showed that the iodine concentration of salt consumed by households in Zhejiang decreased from 28.80 mg/kg in 2010 to 22.08 mg/kg in 2022. The value in 2022 still met the old standard in 2000 [24] and the new standard implemented in 2012 [15]; however, the median salt iodine concentration in coastal regions in 2022 was only 0.42 mg/kg, far below the national standard. Furthermore, the consumption rate of qualified iodized salt in Zhejiang decreased from 76.65% in 2010 to 64.20% in 2022, which was significantly <90%, the value specified by the national standard for IDD elimination [25]. Furthermore, the large interregional differences indicated that some residents gave up iodized salt; hence, the risk of iodine deficiency increased, especially in coastal regions.

The dietary iodine intake among residents in Zhejiang in 2022 was 142.05 μg/d. This was between the recommended nutrient intake (RNI) and the tolerable UL established by the Chinese Nutrition Society; however, it was significantly lower than that (277.48 μg/d) in 2010. This may have been due to the reduction in iodine concentration of salt in China in 2012 and the reduction in the consumption rates of qualified iodized salt. This finding is consistent with the results of a study in Fujian [26]. The two surveys coincidently showed that the dietary iodine intake differed significantly among residents in different regions (inland regions > sub-coastal regions > coastal regions), and the intake in each region was positively correlated with the consumption rate of qualified iodized salt in that region. The dietary iodine intake in sub-coastal and inland regions was sufficient and safe; however, the intake among residents in coastal areas was insufficient, requiring public health interventions. However, no significant difference was observed in dietary iodine intake between urban and rural areas, likely due to the implementation of USI and increased health education, suggesting the effectiveness and equity of iodine nutrition interventions [27].

Moreover, the proportion of residents with dietary iodine intake below the EAR increased from 15.10% to 34.80%, while the proportion of residents with dietary iodine intake above the tolerable UL decreased from 15.00% to 2.90%. These results indicate that the possible health risks caused by iodine deficiency in Zhejiang at this stage may be much greater than those caused by iodine excess. Reportedly, the dietary iodine intake in Fujian, China was below the EAR in 7.4% of adults and higher than the tolerable UL in 1.5% [26].

Salt is still the main source of dietary iodine intake in Zhejiang, and this result is consistent with the findings of recent studies conducted in other parts of China [13,28]. However, the contribution rate of salt to dietary iodine intake decreased from 73.45% to 48.54%, which could be attributed to the decline in the consumption rate of qualified iodized salt and the reduction in salt iodine concentrations. With the success recorded in IDD prevention and treatment, some serious iodine deficiency-related diseases with obvious symptoms, such as cretinism and goiter, have been greatly reduced. Consequently, residents’ awareness of IDD prevention and treatment has gradually weakened [29]. In particular, some residents believe that since Zhejiang is a coastal region, there is no need to supplement iodine because of the frequent consumption of iodine-rich seafood. As a result, the residents are reluctant to consume iodized salt; hence, there is a gradual decline in the consumption rate of qualified iodized salt. Furthermore, reducing iodine concentration in salt will inevitably lead to a decrease in iodine intake from salt, thereby reducing the contribution rate of salt to dietary iodine intake.

Compared with 2010, the contribution rates of *Porphyra* and salt to dietary iodine intake in 2022 decreased significantly, while the contribution rates of drinking water, kelp, other seafood, and other foods increased significantly. These changes may be attributed to the changes in dietary patterns. In recent years, the government and relevant agencies have taken the lead in issuing various healthy dietary guidelines [30]. These guide residents in reducing salt and oil intake and increasing the proportion of whole grains, vegetables, fruits, and dairy products in food intake. These recommendations have also affected the dietary patterns of the population. In addition, since the sum of all contribution rates is always 100%, the contribution rates of drinking water and other foods will naturally increase in response to the decline in the contribution rate of salt.

Kelp and *Porphyra* are rich in iodine, but their contribution to dietary iodine intake is limited due to their low consumption frequency and volume. In contrast, the contribution to dietary iodine intake by other seafood consumed more frequently is also limited because of their low iodine concentrations. Hence, as the iodine intake from salt is decreasing, its intake from drinking water and food alone is far from meeting the normal needs of the human body. Notably, the contribution rate of salt in dietary iodine intake was lower in coastal regions than in other regions. In contrast, the contribution rate of *Porphyra* and other seafood in dietary iodine intake was higher in coastal regions than in other regions. These results indicate that more residents in coastal regions gave up iodized salt. Furthermore, although the contribution rate of seafood such as *Porphyra* in coastal regions is relatively high due to rich sources, the dietary iodine intake in coastal regions is still lower than that in inland and sub-coastal regions, which further indicates that seafood is not the main source of dietary iodine in Zhejiang.

Zhejiang needs to improve the policy of salt iodine supplementation further and adopt reliable iodine supplementation measures. For example, relevant laws and regulations for protecting the health of the general public can be formulated to clarify the standards for producing and selling iodized salt, ensuring that the policies have laws to follow. An iodine nutrition surveillance system should be established to regularly carry out surveys on the iodine nutrition status of residents to assess the consumption rate of iodized salt and adjust the iodine supplementation strategy promptly. It is also necessary to implement differentiated iodized salt strategies according to different regions’ iodine concentrations in the natural environment. Moreover, there is also a need to improve medical personnels’ knowledge of iodine and other nutrients, especially in areas with relatively low education levels. Furthermore, various channels should be utilized to spread awareness of the adverse effects of IDDs and the importance of iodized salt and the correct use of it, thus enhancing the public’s awareness of self-care. For pregnant and lactating women, children, and other special groups with high iodine needs, special iodine supplementation guidelines and measures should be formulated, and requisite nutritional guidance and services should be provided.

In this study, dietary surveys were used to compare the dietary iodine status of residents in Zhejiang in 2010 and 2022 to analyze the changes in dietary iodine intake and its sources. However, this study had a few limitations. For example, the iodine concentrations of various foods used in this study were obtained from the China Food Composition; however, the composition of food from different origins may differ. Therefore, the dietary iodine intake calculated in this study may not be completely accurate. In addition, the dairy market in China is experiencing growth, and the purchase and consumption of milk in Zhejiang Province significantly influenced iodine intake. Given the varying iodine content in milk and the dietary habits of the population, we did not conduct a separate analysis of dairy products in this study. This can be considered in future research.

## 5. Conclusions

In summary, at this stage, the dietary iodine intake of residents in Zhejiang showed a downward trend, although generally adequate. However, the dietary iodine intake among residents in coastal regions was obviously insufficient. The salt iodine concentration and the consumption rate of qualified iodized salt in households in Zhejiang had significantly decreased from 2010 to 2022. The changes in residents’ dietary patterns led to changes in the sources of dietary iodine. Salt remained the main source of dietary iodine; however, its contribution to dietary intake decreased significantly. Therefore, Zhejiang must continue to implement comprehensive IDD prevention and control measures based on salt iodization and reverse the trend of the continuous decline in the consumption rate of qualified iodized salt through various means such as health education to protect the health of residents and prevent the resurgence of IDDs.

## Figures and Tables

**Table 1:** Reference dietary iodine intake standards for Chinese residents.

Age/Stage	EAR	RNI	UL
0~	-	85 *	-
0.5~	-	115 *	-
1~	65	90	-
4~	65	90	200
7~	65	90	300
11~	75	110	400
14~	85	120	500
18~	85	120	600
Pregnancy	160	230	600
Lactation	170	240	600

* Adequate intake (Al); references for the estimated average requirement (EAR), recommended nutrient intake (RNI), and tolerable upper intake level (UL) for iodine were obtained from the “Dietary Reference Intakes for Chinese Residents”.

**Table 2:** Basic information of survey respondents in 2010 and 2022.

Characteristics	2010		2022	
	Number of People	Proportion (%)	Number of People	Proportion (%)
Age				
<18	1574	16.10	868	14.70
18–44	3646	37.20	1713	29.10
45–59	2710	27.70	1636	27.80
≥60	1868	19.10	1673	28.40
Sex				
Male	4752	48.50	2734	46.40
Female	5046	51.50	3156	53.60
Region				
Coastal	3551	36.20	2106	35.80
Sub-coastal	3530	36.00	2031	34.50
Inland	2717	27.70	1753	29.80
Urban/Rural areas				
Urban	4849	49.50	3128	53.10
Rural	4949	50.50	2762	46.90
Consumption of iodized salt				
Yes	8909	90.90	3865	65.60
No	889	9.10	2025	34.40
Total	9798	100.00	5890	100.00

**Table 3:** The consumption rates of qualified iodized salt and the iodine concentrations of salt consumed by households in Zhejiang in 2010 and 2022.

Characteristics	2010			2022			*p*-Value
	*N*	Salt Iodine Concentration *M* (*P_25_*_,_ *P_75_*) (mg/kg)	Consumption Rate of Qualified Iodized Salt (%)	*N*	Salt iodine Concentration *M* (*P_25_*_,_ *P_75_*) (mg/kg)	Consumption Rate of Qualified Iodized Salt (%)	
Region							
Coastal	3550	25.19 (0.00, 30.20)	54.96 (1951)	970	0.42 (0.00, 22.70)	37.00 (359)	<0.001
Sub-coastal	2281	29.00 (26.60, 31.70)	93.20 (2126)	918	22.27 (0.00, 24.61)	67.80 (622)	<0.001
Inland	1980	32.40 (28.94, 36.30)	96.46 (1910)	1159	23.42 (21.04, 25.12)	84.20 (976)	<0.001
Urban/Rural areas							
Urban	3845	29.00 (24.50, 32.60)	80.03 (3077)	1501	21.90 (0.00, 24.57)	59.70 (896)	<0.001
Rural	3966	28.50 (13.90, 32.29)	73.37 (2910)	1546	22.20 (1.59, 24.50)	68.60 (1061)	<0.001
Total	7811	28.80 (22.93, 32.40)	76.65 (5987)	3047	22.08 (0.00, 24.51)	64.20 (1957)	<0.001

*N*: sample size; *M*: 50th percentile, i.e., median; *P_25_*: 25th percentile, i.e., the 1st quartile; *P_75_*: 75th percentile, i.e., 3rd quartile. The numerical values outside the parentheses denote the proportions (%), and the numerical values inside the parentheses denote the number of iodine salt samples.

**Table 4:** Dietary iodine intake in Zhejiang in 2010 and 2022.

Characteristics	2010		2022		*Z*-Value	*p*-Value
	*N*	*M* (*P_25_*_,_ *P_75_*) (μg/d)	*N*	*M* (*P_25_*_,_ *P_75_*) (μg/d)		
Region						
Coastal	3551	220.61 (46.21, 390.54) ^a^	2106	75.66 (36.94, 172.02) ^a^	−21.745	<0.001
Sub-coastal	3530	259.24 (170.65, 396.23) ^b^	2031	153.42 (81.35, 251.47) ^b^	−26.347	<0.001
Inland	2717	358.12 (242.81, 511.12) ^c^	1753	185.61 (118.13, 282.17) ^c^	−28.144	<0.001
*H*-value		639.175		588.592		
*p*-value ^1^		<0.001		<0.001		
Urban/Rural areas						
Urban	4949	277.63 (160.15, 425.09)	3128	136.00 (55.14, 244.50)	−31.989	<0.001
Rural	4849	277.32 (150.32, 435.48)	2762	145.89 (63.58, 230.63)	−28.05	<0.001
*Z*-value		−1.591		−1.249		
*p*-value ^2^		0.112		0.211		
Consumption of iodized salt						
Yes	8909	292.70 (185.10, 429.56)	3638	191.98 (133.47, 283.93)	−28.992	<0.001
No	889	37.53 (28.34, 57.29)	2252	42.21 (26.33, 67.77)	−4.186	<0.001
*Z*-value		−38.501		−52.246		
*p*-value ^2^		<0.001		<0.001		
Total	9799	277.48 (155.50, 430.66)	5890	142.05 (58.94, 237.11)	−42.408	<0.001

*M*: 50th percentile, i.e., median; *P_25_*: 25th percentile, i.e., the 1st quartile; *P_75_*: 75th percentile, that is the 3rd quartile; ^1^ *p*-value of the Kruskal–Wallis *H* test; ^2^ *p*-value of the Mann–Whitney *U* test; ^a,b,c^ subgroups with different letters indicate significant differences (*p* < 0.05).

**Table 5:** The distribution of residents with dietary iodine intake above or below the reference intake values in Zhejiang in 2010 and 2022.

Year	Characteristics	N	<EAR	EAR–RNI	RNI–UL	>UL	*χ* ^2^	*p*-Value
2010	Total	9798	1478 (15.10)	365 (3.70)	6489 (66.20)	1466 (15.00)	1209.798	<0.001
	Coastal	3551	1094 (30.80)	156 (4.40)	1821 (51.30)	480 (13.50)		
	Sub-coastal	3530	237 (6.70)	156 (4.40)	2701 (76.50)	436 (12.40)		
	Inland	2717	147 (5.40)	53 (2.00)	1967 (72.40)	550 (20.20)		
	χ^2^_trend_		849.457	23.278	351.809	48.115		
	P_for trend_		<0.001	<0.001	<0.001	<0.001		
2022	Total	5890	2050 (34.80)	587 (10.00)	3082 (52.30)	171 (2.90)	723.632	<0.001
	Coastal	2106	1155 (54.80)	184 (8.70)	719 (34.10)	48 (2.30)		
	Sub-coastal	2031	614 (30.20)	237 (11.70)	1147 (56.60)	33 (1.60)		
	Inland	1753	281 (16.00)	166 (9.50)	1216 (69.40)	90 (5.10)		
	χ^2^_trend_		647.777	0.857	485.333	25.229		
	P_for trend_		<0.001	0.355	<0.001	<0.001		

The numerical values outside the parentheses denote the number of people, and those inside the parentheses denote the proportions (%). References for the estimated average requirement (EAR), recommended nutrient intake (RNI), and tolerable upper intake level (UL) for iodine were obtained from the “Dietary Reference Intakes for Chinese Residents”.

**Table 6:** Contribution rates of different food sources to dietary iodine intake in residents in Zhejiang in 2010 and 2022.

Category		*N*	Contribution Rate (μg/d, %)					
			Salt	Drinking Water	*Porphyra*	Kelp	Other Seafood *	Other Foods **
Total	2010	9798	73.45 (250.03)	1.39 (4.74)	16.46 (56.02)	1.17 (3.98)	1.27 (4.33)	6.26 (21.32)
	2022	5890	48.54 (107.42)	8.84 (6.52)	4.82 (15.99)	3.02 (13.77)	2.31 (2.05)	32.46 (35.08)
	*p*-value		<0.001	<0.001	<0.001	0.004	<0.001	<0.001
Coastal	2010	3551	65.37 (189.73)	1.59 (4.62)	23.57 (68.42)	1.13 (3.28)	2.48 (7.19)	5.85 (16.99)
	2022	2106	30.95 (60.94)	12.73 (7.06)	7.14 (18.01)	3.61 (12.92)	4.03 (3.23)	41.55 (32.67)
	*p*-value		<0.001	<0.001	<0.001	0.623	0.113	<0.001
Sub-coastal	2010	3530	73.01 (252.99)	1.76 (6.09)	16.99 (58.88)	0.95 (3.28)	0.69 (2.39)	6.60 (22.87)
	2022	2031	51.61 (116.03)	10.89 (10.13)	3.80 (12.67)	1.34 (10.71)	1.75 (1.58)	30.61 (37.86)
	*p*-value		<0.001	<0.001	0.162	<0.001	0.099	<0.001
Inland	2010	2717	81.64 (325.00)	0.79 (3.16)	9.06 (36.08)	1.45 (5.79)	0.78 (3.10)	6.27 (24.97)
	2022	1753	66.11 (153.30)	1.80 (1.70)	3.23 (17.39)	4.27 (18.33)	0.90 (1.18)	23.69 (23.56)
	*p*-value		<0.001	<0.001	<0.001	0.149	<0.001	<0.001
Urban	2010	4949	68.75 (239.81)	6.12 (6.83)	7.57 (67.57)	1.61 (4.24)	2.77 (4.55)	13.18 (23.98)
	2022	3128	46.23 (104.89)	9.22 (5.99)	4.93 (16.79)	2.81 (8.42)	3.18 (2.82)	33.63 (37.98)
	*p*-value		<0.001	<0.001	<0.001	<0.001	0.714	<0.001
Rural	2010	4849	69.77 (275.52)	7.99 (5.92)	4.81 (44.23)	1.51 (3.70)	2.41 (4.10)	13.50 (18.60)
	2022	2762	51.16 (110.30)	8.41 (7.12)	4.71 (15.08)	3.26 (19.83)	1.33 (1.18)	31.14 (31.80)
	*p*-value		<0.001	<0.001	0.316	0.684	<0.001	<0.001

The numerical values inside parentheses denote the median iodine: intake (μg/d) from food, salt, and drinking water; * Other seafood: including marine fish, shrimp, crab, shellfish; ** Other foods: including cereals, potatoes, dried beans, vegetables, fruits, meat, dairy, eggs, freshwater fish, and so on.

## Data Availability

The data presented in this study are available on request from the corresponding author. The data are not publicly available due to confidentiality concerns.
